# Correction: Flynn et al. Introducing and Familiarising Older Adults Living with Dementia and Their Caregivers to Virtual Reality. *Int. J. Environ. Res. Public Health* 2022, *19*, 16343

**DOI:** 10.3390/ijerph21070911

**Published:** 2024-07-12

**Authors:** Aisling Flynn, Marguerite Barry, Wei Qi Koh, Gearóid Reilly, Attracta Brennan, Sam Redfern, Dympna Casey

**Affiliations:** 1 School of Nursing and Midwifery, University of Galway, H91 TK33 Galway, Irelanddympna.casey@universityofgalway.ie (D.C.); 2 Information and Communication Studies, ADAPT Centre, University College Dublin, D04 V1W8 Dublin, Ireland; 3 School of Computer Science, University of Galway, H91 TK33 Galway, Ireland; g.reilly6@universityofgalway.ie (G.R.); sam.redfern@universityofgalway.ie (S.R.)

The authors would like to make the following corrections to the published article [1]. There was miscommunication between the authors and the production team during the article production process. As such, several editorial changes (primarily language-related revisions) requested during proofreading were not reflected in the paper published on 16 December 2022. The language revisions are as follows:

## 1. Error in Figures and Tables

The following language mistakes in Figures 2 and 5 in the original publication were corrected:

- Caption changed from “television” to “a television” (Figure 2, p. 5).
- “SP” changed to “caregiver” (Figure 5, p. 11).
- Caption changed to “themes and corresponding subthemes” (Figure 5, p. 11).

The corrected Figure 5 appears below.

**Figure 5:**
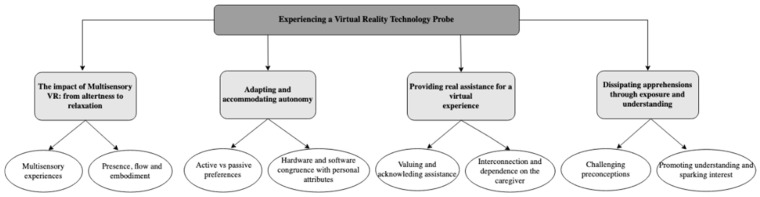
Thematic map of findings illustrating the overarching themes and corresponding subthemes.

The following language mistakes in Table 5 in the original publication were corrected:

- “Encorporate” changed to “Incorporate” (p. 17).
- “real-time” changed to “real time” (p. 17).
- “Providing” changed to “Provide” (p. 17).
- “dementia and caregiver” changed to “dementia and caregivers” (p. 18).
- “set up” changed to “set-up” (p. 18).
- “Acknowledging” changed to “Acknowledge” (p. 18).
- “Discuss people living with dementias and caregivers presumptions of VR before use” changed to “Discuss the presumptions of VR with people living with dementia and their caregivers before use” (p. 18).
- “Have caregiver present during people living with dementia for reassurance and support” changed to “Have the caregiver present when people living with dementia are using VR for reassurance and support” (p. 18).
- “for the people living with dementia” change to “for people living with dementia”

## 2. Addition of an Author’s ORCID

ORCID ID for one author (Dympna Casey) was not included in the original publication. The ORCID ID has been updated.

## 3. Amended References

Full-text reference [9] has been corrected in the reference section to:

Koh, W.Q.; Heins, P.; Flynn, A.; Mahmoudi, A.; Garcia, L.; Malinowsky, C.; Brorsson, A. Bridging gaps in the design and implementation of socially assistive technologies for dementia care: The role of occupational therapy. *Disabil. Rehabil. Assist. Technol.* **2022**, 1–9. https://doi.org/10.1080/17483107.2022.2111610.

Some in-text citations have been shortened: Abeele et al. [54] (Section 2.2, Para 6, p. 4), Goodall et al. [67] (Section 4, Para 3, p. 15), Karaosmanoglu et al. [55] (Section 4, Para 4, p. 15, Section 4, Para 6, p. 15), Koh et al. [9] (Section 4, Para 5, p. 15) and Waycott et al. [75] Section 4, Para 7, p. 15.

## 4. Text Corrections

There was inconsistent use of UK and US spelling conventions in the original publication. The following changes were made to ensure consistent UK spelling:

- “pre-requisite” to “prerequisite” (abstract, p1; Section 3, para 1, p. 11; Section 7, para 1, p. 18).
- “individualized” to “individualised” (Section 1, para 1, p. 1; Section 3.3, para 1, p. 12)
- “toward” to “towards” (Section 1, para 2, p. 2).
- “prioritized” to “prioritised” (Section 1, para 3, p. 2).
- “familiarize” to “familiarise” (Section 1, para 4, lines 3 and 10, p. 2).
- “utilized” to “utilised” (Section 2.2, para 5, p. 4).
- “trialing” to “trialling” (Section 2.1, para 2, p. 3; Section 2.4, para 1, p. 8).
- “fieldnotes” to “field notes” (Section 2.5, para 1, p. 9; Section 2.7, para 1, p. 10, Section 3.1, para 2, p. 11, Section 3.3, para 4, p. 14).
- “minimize“ to “minimise” (Section 2.6, para 1, p. 9; Section 2.8, para 3, p. 10).
- “familiarizing” to “familiarising” (Section 2.7, para 1, p. 10).
- “rigor” to “rigour“ (Section 2.7, para 3, p. 10).
- “person-centered” to “person-centred” (Section 2.8, para 3, p. 10).
- “verbalizations” to “verbalisations” (Section 3.1, para 1, p. 11; Section 4, para 2, p. 14).
- “personalization” to “personalisation” (Section 3.1, para 1, p. 11).
- “swiveling” to “swivelling” (Section 3.2, para 2, p. 12).
- “familiarization” to “familiarisation” (Section 3.2, para 4, p. 12).
- “real-time” to “real time” (Section 3.3, para 2 and 4, p. 13).
- “mobilization” to “mobilisation” (Section 4, para 9, p. 16).

Inconsistencies regarding the use of singular and plural instances were corrected:

- “individuals” to “individual’s” (Section 1, para 3, p. 2).
- “caregiver” to “caregivers” (Section 1, para 4, p. 2; Section 1, para 6, p. 3; Section 2.1, para 2, lines 3 and 6, p. 3; Section 2.2, para 5, p. 4; Section 2.3, para 1, p. 7; Section 2.3, para 2, p. 7; Section 2.5, para 1, line 11, p. 9; Section 2.6, para 1, line 1, para 2, lines 1 and 9, p. 9; Section 3.1, para 1, lines 2 and 12, p. 11; Section 3.2, para 1, p. 12; Section 3.2, para 3, p. 12; Section 3.2, para 4, lines 2 and 14, p. 12; Section 3.3, para 3, lines 7 and 12, p. 13; Section 4, para 6, line 9, p. 15; Section 4, para 9, p. 16; Section 6, para 1, p. 18).
- “Having the caregiver involved” to “Having caregivers involved” (Section 2.3, para 1, line 7, p. 7).
- “caregivers” to “caregivers’” (Section 2.5, para 1, lines 5 and 12, p. 9).
- “dementias’” to “dementia’s” (Section 2.5, para 1, line 7, p. 9).
- “environment” to “environments” (Section 2.6, para 1, p. 9).
- “caregiver” to “their caregivers’” (Section 2.7, para 2, p. 10).
- “VE’s” to “VEs” (Section 3.1, para 1, p. 11; Section 6, para 1, p. 18).
- “of the people living with dementia” to “people living with dementia” (Section 3.2, para 2, line 9, p. 12, Section 3.3, para 1, p. 12; Section 3.3, para 3, p. 13; Section 3.3, para 5, p. 13; Section 3.4, para 1, p. 14; Section 4, Para 5, p. 15; Section 4, Para 6, p. 15).
- “the caregiver’s” to “their caregiver’s” (Section 3.3, para 4, line 4, p. 12).
- “as a co-facilitator” to “as co-facilitators” (Section 3.3, para 5, p. 13).
- “OT’s” to “OTs” (Section 4, para 5, p. 15).
- “caregivers” to “their caregivers’” (Section 4, para 8, p. 16).
- “experience” to “experiences” (Section 4, para 8 and 9, p. 16).
- “was” to “were” (Section 4, para 9, p. 16)

Spelling revisions, revisions to tenses and punctuations, and sentence revisions were requested during the initial proofreading stage to improve the clarity of the manuscript. As these changes were not reflected in the original publication, they have now been corrected:

- “and facilitate” to “and can facilitate” (Section 1, para 2, p. 2).
- “and inspire” to “and can inspire” (Section 1, para 4, p. 2).
- “generalfunctionality” to “general functionality” (Section 1, para 5, p. 2).
- “well-being” to “wellbeing” (Section 2.1, para 2, p. 3).
- “consists” to “consisted” (Section 2.2, para 1, p. 3).
- “Furthermore, the lead researcher, who is a qualified Occupational Therapist (OT), using her professional judgement considered the visual and auditory content of commercial active alternatives to be overly stimulating and complex, and the interactions within VR were not adaptable which contradicted the open-ended and exploratory nature of technology probes proposed in the literature [42].” amended to “Furthermore, the lead researcher, who is a qualified Occupational Therapist (OT), using her professional judgement, considered the visual and auditory content of commercial active alternatives to be overly stimulating and complex. In addition, the interactions within VR were not adaptable, which contradicts the open-ended and exploratory nature of technology probes proposed in the literature” (Section 2.2, para 2, p. 4).
- “PAR group were” to “PAR group was” (Section 2.3, para 1, p. 7).
- “except for one [PwD7] who turned 60 years during the project” changed to “except for one person living with dementia who turned 60 years during the project” (Section 2.3, para 2, p. 7).
- “In terms of VR experience, five people living with dementia and their caregivers stated they had seen VR but not used it personally, seven people living with dementia and their caregiver reported they had previously tried VR albeit, several years previous and only passively through less interactive headsets, whilst six people living with dementia and their caregivers stated that they had no prior knowledge of VR” amended to “In terms of VR experience, five people living with dementia and their caregivers stated they had seen VR but not used it personally, seven people living with dementia and their caregiver had tried passive VR (involving less interactive headsets) several years ago, whilst six people living with dementia and their caregivers stated that they had no prior knowledge of VR” (Section 2.4, para 2, p. 8).
- Bracket added “(Supplementary S3 and S4)” (Section 2.5, para 1, p. 9).
- “important; the” to “important, the” (Section 2.5, para 1, p. 9).
- “It also” to “This approach also” (Section 2.7, para 3, p. 10).
- “care settings, and” to “care settings and” (Section 2.8, para 1, p. 10).
- “They explored participants’ experiences beyond their diagnoses of dementia to explore the interplay between the physical, mental, and social functioning of people living with dementia and how these influenced their experience of VR” changed to “They explored people with dementia’s experiences beyond their diagnoses, to explore the interplay between their physical, mental, and social functioning and how these influenced their experience of VR” (Section 2.8, para 2, p. 10).
- “data..” changed to “data.” (Section 2.8, para 2, line 6, p. 10).
- Removed duplicate sentence “To minimise researcher bias, a summary of the finding was presented to people living with dementia and their caregivers [64].” (Section 2.8, para 3, line 5, p. 10).
- “was alluded” to “were alluded” (Section 3.1, para 2, p. 11).
- “In contrast to positive” to “In contrast to the positive” (Section 3.1, para 4, p. 12)
- Removed “[REF]” (Section 3.1, para 4, p. 12).
- “the people living with dementia” to “people living with dementia” (Section 2.5, para 1, p. 9; Section 3.1, para 1, p. 11; Section 3.3, para 3, p. 13, Section 3.4, para 1, p. 14, Section 4, Para 6, p. 15, Section 4, Para 9, p. 16).
- “peopleliving” to “people living” (Section 3.3, para 1, p. 12).
- “capabilities “It’s” to “capabilities, “It’s” (Section 3.3, para 3, p. 13).
- “ensured the abilities” to “ensured that the abilities” (Section 3.3, para 3, p. 13).
- “Three people living with dementia verbalized that the presence of the caregiver was useful” to “Three people living with dementia verbalised that their caregiver’s presence was useful” (Section 3.3, para 4, p. 13).
- Removed “[1,3]” (Section 3.3, para 4, p. 14).
- Removed “set up the” (Section 3.3, para 5, line 4, p. 13).
- “the preferences of the people living with dementia but, ” to “the preferences of not only people living with dementia but” (Section 3.3, para 5, p. 13).
- “both of” to “all of” (Section 3.3, para 5, p. 14).
- considered as “gamers”” to “considered gamers” (Section 3.4, para 1, p. 14).
- “people living with dementia and their caregivers’” to “people living with dementia and their caregiver’s” (Section 3.4, para 1, line 14, p. 14).
- “:” to “,” (Section 3.4, para 1, line 6, p. 14).
- “Understanding VR sparked interest in its future use for people living with dementia [1–9]” changed to “Understanding VR sparked interest in its future use for people living with dementia” (Section 3.4, para 2, p. 14).
- “the VR FOUNDations” changed to “VR FOUNDations” (Section 4, para 2, line 1, p. 14).
- “compliments” to “complements” (Section 4, para 5, p. 15).
- “acknowledgment” to “acknowledgement” (Section 4, para 5, p. 15).
- “fostered autonomous” to “fostered the autonomous” (Section 4, para 5, p. 15).
- “and which acknowledges people living with dementia’s wider sociotechnical system influencing VR implementation” to “and acknowledges the wider sociotechnical system influencing VR implementation for people living with dementia” (Section 4, para 6, p. 15).
- “advocated” to “advocate” (Section 4, para 7, p. 15).
- “of bespoke VR application” to “of a bespoke VR application” (Section 4, para 8, p. 16).
- “present” to “lead to” (Section 5, para 1, p. 17).
- “presented in” to “; these are presented in” (Section 5, para 2, p. 17).
- “Outside of informing technology probe research, the findings can influence broader VR design research. This study provides valuable insights into how a VR technology probe may be used in dementia care research” changed to “Outside of informing technology probe research, the findings also influence broader VR design research by providing valuable insights into how a VR technology probe may be used in dementia care research.” (Section 5, para 2, p. 17).
- Removed “In”” (Institutional Review Board Statement, p. 19)

Errors in the placement of commas have been corrected (removed, added or moved):

- “Gaver et al. [36] technology” to “Gaver et al. [36], technology” (Section 1, para 4, p. 2).
- “VEs, is” to “VEs is” (Section 1, para 4, p. 2).
- “thus,” to “, thus” (Section 1, para 5, p. 3).
- “dementia, and” to “dementia and” (Section 2.1, para 1, p. 3).
- “Games Developer, iteratively” to “Games Developer iteratively” (Section 2.2, para 4, p. 4).
- “dementia, who” to “dementia who” (Section 2.2, para 4, p. 4).
- “advisory group participated” to “advisory group, participated” (Section 2.2, para 4, p. 4).
- “VR FOUNDations, consisted” to “VR FOUNDations consisted” (Section 2.2, para 5, p. 4).
- “who” to “, who” (Section 2.2, para 6, p. 4; Section 2.4, para 1, p. 8; Section 4, para 3, line 5 and 10, p. 15; Section 4, para 7, p. 16).
- “whereby,“ to “, whereby” (Section 2.3, para 2, p. 7; Section 3.2, para 2, p. 12; Section 3.4, para 1, p. 14).
- “with” to “, with” (Section 2.4, para 1, p. 8).
- “analysis” to “analysis,” (Section 3, para 1, p. 11).
- “albeit” to“, albeit” (Section 3.1, para 1, p. 11; Section 4, para 9, p. 16).
- “functioning” to “functioning,” (Section 3.2, para 2, p. 12).
- “set up” to “set-up,” (Section 3.3, para 5, line 3, p. 13).
- “while,” to “, while” (Section 3.4, para 1, p. 14).
- “probes” to “probes,” (Section 4, para 1, p. 14).
- “caregivers, but for” to “caregivers but for” (Section 4, para 1, p. 14).
- “It is noteworthy, that” to “It is noteworthy that” (Section 4, para 3, p. 15).
- “responses” to “responses,” (Section 4, para 3, lines 4, p. 15).
- “and in particular,” to “, and in particular” (Section 4, para 8, p. 16).
- “for most,” to “, for most” (Section 4, para 8, p. 16).
- “by using this approach” to “by using this approach,” (Section 4, para 9, p. 16).
- “which” to “, which” (Section 4, para 9, p. 16; Section 5, para 1, lines 7 and 10, p. 17).
- “caregivers and” to “caregivers, and” (Section 5, para 1, p. 17).

## 5. Reporting of Findings

In the findings section of the original publication, referencing cases (i.e., [PwD X,Y,Z; CG X,Y,Z]) has been removed to increase the readability, accessibility and flow of the findings section. Case references were not removed for direct participant quotations (Section 3, pp. 11–14).

## 6. Acknowledgements

“The contribution of David Healy, School of Psychology, University of Galway in informing the design of VR FOUNDations would also like to be acknowledged” replaced with: “We would like to acknowledge David Healy, School of Psychology, University of Galway, for his contributions to the design of VR FOUNDations”.

The authors apologise for any inconvenience caused and state that the scientific conclusions are unaffected. This correction was approved by the Academic Editor. The original publication has also been updated.
