# Supplementary material for: Introducing and Familiarising Older Adults Living with Dementia and Their Caregivers to Virtual Reality
Source: Int J Environ Res Public Health. 2022 Dec 6;19(23):16343. doi: 10.3390/ijerph192316343 (PMC9736737; doi:10.3390/ijerph192316343)
Supplement: Supplementary file 1 [file ijerph-19-16343-s001.zip › ijerph-1980438-supplementary_AF.pdf]

**S1. Detailed Demographic Data**

| Cases | Gender | Age Range | Marital Status | Current Employment Status | Length of Memory Problems | Current primary caregiver | Highest Level of Education |
|---|---|---|---|---|---|---|---|
| PwD1 | Male | 59-69 | Married/Domestic Partnership | Retired | 1-3 years | Spouse/Partner | Third Level Degree |
| PwD2 | Male | 59-69 | Married/Domestic Partnership | Retired | 4-6 years | Spouse/Partner | Professional Qualification |
| PwD3 | Male | 70-79 | Married/Domestic Partnership | Retired | 4-6 years | Daughter | Secondary Education |
| PwD4 | Male | 70-79 | Married/Domestic Partnership | Retired | 1-3 years | Daughter | Secondary Education |
| PwD5 | Female | 80+ | Widowed | Retired | 7+ years | Daughter | Third Level Degree |
| PwD6 | Male | 59-69 | Married/Domestic Partnership | Retired | 1-3 years | Spouse/Partner | Third Level Degree |
| PwD7 | Male | 70-79 | Married/Domestic Partnership | Retired | 1-3 years | Spouse/Partner | Professional Qualification |
| PwD8 | Male | 59-69 | Married/Domestic Partnership | Retired | 4-6 years | Spouse/Partner | Secondary Education |
| PwD9 | Female | 70-79 | Married/Domestic Partnership | Retired | 1-3 years | Daughter | Professional Qualification |

| Cases | Gender | Age Range | Marital status | Employment Status | Relationship to PwD | Length of time supporting PwD | Education Level |
|---|---|---|---|---|---|---|---|
| CG1 | Female | 50-59 | Married/Domestic Partnership | Self-employed | Spouse/Partner | 0-4 years | Third Level Degree |
| CG2 | Female | 50-59 | Married/Domestic Partnership | Employed | Spouse/Partner | 5-9 years | Professional Qualification |
| CG3 | Female | 40-49 | Single | Other | Father | 0-4 years | Third Level Degree |
| CG4 | Female | 40-49 | Single | Employed | Father | 0-4 years | Third Level Degree |
| CG5 | Female | 50-59 | Single | Employed | Mother | 5-9 years | Third Level Degree |
| CG6 | Female | 50-59 | Married/Domestic Partnership | Retired | Spouse/Partner | 0-4 years | Professional Qualification |
| CG7 | Female | 60-69 | Married/Domestic Partnership | Retired | Spouse/Partner | 0-4 years | Third Level Degree |
| CG8 | Female | 60-69 | Married/Domestic Partnership | Self-employed | Spouse/Partner | 5-9 years | Post Leaving Cert |
| CG9 | Female | 30-39 | Married/Domestic Partnership | Employed | Mother | 0-4 years | Third Level Degree |

**S2. Observational Field Note Template**

Participant ID:
Date:
Time Started:
Time ended:
Support person present:
Researcher present:

| Observation Prompt | Notes | Time(s) experienced |
|---|---|---|
| Positive response (verbal and non-verbal)<br>- Initiate conversation or make vocalization that shows interest (For example, ooh's, ah's, giggling, or saying 'wow')<br>- Smiling, laughing<br>- Verbally expressing interest<br>Negative response (verbal and non-verbal)<br>- Verbally expressing disinterest<br>- Tearful<br>- Aggression<br>- Restless<br>- Attempting to take the headset off | | |
| Tolerability<br>- Independently adjusting the headset or asking for it to be taken off<br>- Adjusting the controllers<br>- Expressing comfort/discomfort<br>- Expression of dizziness or disorientation<br>- Expression of eye strain<br>- Verbal feedback on HMD or controllers<br>o Expressing ease or dis-ease | | |
| Body positioning<br>- Any changes in body movement<br>- Readjustment of body positioning<br>- Over-reaching | | |
| Support Person or Facilitator role:<br>- Assistance to use the headset, controllers<br>- Verbal assistance during use<br>- Physical assistance during use (e.g positioning, adjusting headset etc.) | | |

**S3. VR Training Environment semi-structured interview schedule (Older adults living with dementia)**

| | |
|---|---|
| **Likability and emotional impact** | 1. What was the experience like?<br>a. What did you like most?<br>b. What did you dislike?<br>c. What would you change?<br>d. How do you feel after using the VR? Sickness etc.<br>Prompts: exciting, stimulating, boring, tiring? |
| **Immersion/Presence** | 1. How did you feel during the experience?<br>a. How did you feel in the room?<br>b. What was the room like?<br>Prompts: Were you aware of your actual room when using the headset? When you had the headset on were you aware of your own kitchen/living room/room on campus? Did you forget about your real surroundings?<br>c. Hands in VR? Did you feel like you were in control of your hands and actions?<br>d. How did you find the length of time? Did it feel too short, too long, just right? What would be a good length of time... like a TV/radio show, phone call or...? |
| **Usability:<br>Perceived Ease of Use (PEOU) and PU** | 1. What did you find easiest to use?<br>Prompts: The controllers? [researcher present controllers]/The headset? [researcher present headset]/Tell me more about that…<br><br>2. What did you find hardest to use?<br>Prompts: The controllers? [researcher present controllers]/The headset? [researcher present headset]/Tell me more about that…<br><br>3. How did you find the instructions relating to the tasks?<br>Prompts: Were they difficult/easy to follow?<br><br>4. Were tasks easy to complete?<br>a. If so, why?<br>b. If not, why?<br>c. Movement in VR/clear tiles<br><br>5. How did you find the weight on your head? [researcher present headset]<br>Prompts: Was it too light? Was it comfortable ; heavy?/Tell me more../How might we fix that?<br><br>6. Would you use this technology?<br>a. If so, why?<br>b. If not, why?<br>Prompts: How could we fix this?<br><br>7. Would this be useful technology for people with dementia/memory difficulties?<br>a. If so, why?<br>b. If not, why?<br><br>8. In your opinion, was it helpful to have my assistance during the session? /In your opinion, was it helpful to have [caregivers name] present during the session? Do you think you could set it up without assistance in the future? |
| **Inspiring future design** | 9. In you opinion, did the experience give you an idea of what VR can do/is like?<br>Prompts: In your opinion, do you think using VR will help you make design decisions in the future? Has it provided you with some ideas of what you might like or not like to see/do for future applications? |

| | |
|---|---|
| | 10. Anything to add? Any design changes or anything obvious that may have been omitted? |

**S4. VR training environment semi-structured interview schedule (Caregiver)**

1. How much assistance was required to [set-up] and use VR with [name of person living with dementia]?
   a. Could you expand on how you assisted with this?
   b. Prompt: do you think your presence was useful for x during the session? Reassurance etc. do you think my presence was useful for x during the session?
2. In your opinion, do you think VR would be difficult to set up (if researcher was not present)?
   a. If so, why?
   b. If not, why?
   c. How could we make set-up easier if you and x had to this independently?
      i. Instruction, videos, etc.
3. From your experience of [name of person living with dementia], how would you gauge their reaction to the technology?
   a. In your opinion, did [name of person living with dementia] enjoy the overall experience?
   b. If yes, why?
   c. If not, why not?
   d. How could we have the experience more accessible for [name of person living with dementia?]
4. In your opinion, was using VR today useful to give you an idea of what it is like?
   a. If so, why?
   b. If not, why?

Prompts: In your opinion, do you think using VR will help you make future design decisions for you and [name of person living with dementia]? Has it provided you with ideas for what you might like or not like to see/do for future applications? Can you give an example?

5. Anything else you would like to add? From your experience of using the technology? Any design changes/obvious omissions?

**S5. Eligibility Checklist**

Participant ID:

Date:

Researcher:

| | Yes | No |
|---|---|---|
| Support-person present for duration of VR-use | | |
| Person with dementia given verbal consent to use the headset (audio recorded) | | |
| Person with dementia stated they do not feel unwell e.g dizziness, flu like symptoms etc. | | |
| Adequate space in environment to facilitate VR | | |

Signed: ________________________

Researcher signature

**S6. Ethical Distress Protocol**

This procedural protocol is to ensure that the wellbeing and rights of the person with dementia are protected. The steps outlined below are for participants' benefit if they become distressed during the data collection process. Before starting the call, the researcher will ensure that the PAR relative is present in the home should the PWMD become distressed.

If a participant becomes distressed or upset during the interview (restlessness, agitation, repetitive questioning, wandering, crying):

1. Ask the person if they would like to take a break (turn off their camera or mute themselves)
   a. If they agree to proceed, the researcher will continue to be vigilant of distress. If the person continues to show signs of distress, the researcher will terminate the interview.
2. Should the person continue to become distressed, ask if they would like to terminate the interview and contact made with the respective relative in the home.

- Before ending the session, ask the participant if they would like the contact numbers for national and local resources. If yes, these can be provided over the call and emailed to themselves or their relatives.
  - Alzheimer Society of Ireland, National helpline: 1800 341 341
  - Alone Ireland: 0818 222024
- The researcher will report the distress to their carer and discuss what needs to be put in place to support the person with dementia, s/he will also inform the PI and the Ethics Advisory and Privacy Board.
